# ZNF750 Is Expressed in Differentiated Keratinocytes and Regulates Epidermal Late Differentiation Genes

**DOI:** 10.1371/journal.pone.0042628

**Published:** 2012-08-24

**Authors:** Idan Cohen, Ramon Y. Birnbaum, Keren Leibson, Ran Taube, Sara Sivan, Ohad S. Birk

**Affiliations:** 1 The Morris Kahn Laboratory of Human Genetics, National Institute for Biotechnology in the Negev, Ben Gurion University, Beer-Sheva, Israel; 2 Department of Molecular Genetics and Virology, Ben-Gurion University, Beer-Sheva, Israel; 3 Department of Bioengineering and Therapeutic Sciences, University of California San Francisco, San Francisco, California, United States of America; 4 Institute for Human Genetics, University of California San Francisco, San Francisco, California, United States of America; 5 Genetics Institute, Soroka Medical Center, Beer-Sheva, Israel; University Hospital Hamburg-Eppendorf, Germany

## Abstract

Disrupted skin barrier due to altered keratinocyte differentiation is common in pathologic conditions such as atopic dermatitis, ichthyosis and psoriasis. However, the molecular cascades governing keratinocyte terminal differentiation are poorly understood. We have previously demonstrated that a dominant mutation in *ZNF750* leads to a clinical phenotype reminiscent of psoriasis and seborrheic dermatitis. Here we show that ZNF750 is a nuclear protein bearing a functional C-terminal nuclear localization signal. ZNF750 was specifically expressed in the epidermal suprabasal layers and its expression was augmented during differentiation, both in human skin and in-vitro, peaking in the granular layer. Silencing of *ZNF750* in Ca2+-induced HaCaT keratinocytes led to morphologically apparent arrest in the progression of late differentiation, as well as diminished apoptosis and sustained proliferation. ZNF750 knockdown cells presented with markedly reduced expression of epidermal late differentiation markers, including gene subsets of epidermal differentiation complex and skin barrier formation such as *FLG*, *LOR*, *SPINK5*, *ALOX12B* and *DSG1*, known to be mutated in various human skin diseases. Furthermore, overexpression of ZNF750 in undifferentiated cells induced terminal differentiation genes. Thus, ZNF750 is a regulator of keratinocyte terminal differentiation and with its downstream targets can serve in future elucidation of therapeutics for common diseases of skin barrier.

## Introduction

Keratinocyte (KC) differentiation is an essential key process in formation and maintenance of the skin barrier. In fact, various common skin diseases such as psoriasis, atopic dermatitis (AD) and ichthyosis involve the disintegration of the epidermal skin barrier due to altered KC differentiation [1]–[3]. The major barrier resides within the exterior layers of the epidermis, which are constantly shed and replaced by inner layer cells that are committed to differentiate and move outwards in a columnar fashion. The epidermis maintains a single basal layer of proliferating cells that adhere to an underlying basement membrane, which retains the ability to self renew under both homeostatic and injury conditions [4]. Upon commitment to terminal differentiation, KCs undergo three distinct differentiation stages, forming the spinous, granular and stratum corneum layers. Each of these phases has specific characteristics in terms of transcription, morphology and function. In the first step of commitment to terminal differentiation, basal cells are withdrawn from the cell cycle, lose their ability to adhere to the basement membrane and migrate to the spinous layer. As cells enter the spinous layer, they switch off the expression of keratin 5 (*KRT*5) and *KRT14* that mark the stratified squamous epithelial cells which possess proliferative potential. Simultaneously, these cells initiate the expression of genes encoding KRT1 and KRT10, which reinforce cell–cell junctions and provide resistance against mechanical stresses at the body surface [5]. At a more advanced stage, granular layer cells begin to express *Filaggrin* (*FLG*) and acquire keratohyalin granules, which contain profilaggrin - the precursor of filaggrin. Filaggrin aggregates the keratin filaments into tight bundles, which collapse the cells into a flattened shape, forming the terminally differentiated cells that comprise the stratum corneum. Simultaneously, other structural genes, including *Involucrin* (IVL), *Loricrin* (LOR) and small proline-rich proteins (SPRRs), are expressed and subsequently crosslinked by transglutaminases to form the chemically resistant cornified envelope structure [6], [7]. In addition, a complex series of lipids, such as ceramides, are synthesized and covalently attached to proteins of the cornified envelope, forming intercellular lamellae that help to produce a complete barrier of the skin. The resulting cornified layer is composed of terminally differentiated, dead, cornified, flattened KC cells that are known as corneocytes [1].

We have previously demonstrated that a dominant mutation in *zinc finger protein 750* (*ZNF750)* causes seborrhea-like dermatitis with psoriasiform elements [8]. The disease phenotype includes dramatically enhanced KC proliferation with parakeratosis, as well as dermal infiltrate of CD4 lymphocytes. The mutation causing this phenotype is within the *ZNF750* coding sequence, abrogating the zinc finger domain of the encoded protein [8]. There is some evidence that ZNF750 might play a role also in bone fide psoriasis since ZNF750 regulatory variants have been identified in classic forms of psoriasis [9]. ZNF750 encodes a novel C2H2 zinc finger protein that is highly expressed in human KCs, but not in dermal fibroblasts or CD4 leukocytes [8], suggesting that the human phenotype induced by *ZNF750* mutation stems from a primary defect in KCs. In the present work, studying both normal and ZNF750 silenced KCs, we determined that ZNF750 is a nuclear protein and characterized its expression pattern. Silencing of ZNF750 effected KCs terminal differentiation in terms of morphology, proliferation and gene expression.

## Results

### ZNF750 is Localized to the Nucleus as Determined by Its C-terminal Nuclear Localization Signals (NLS)

**Figure 1:**
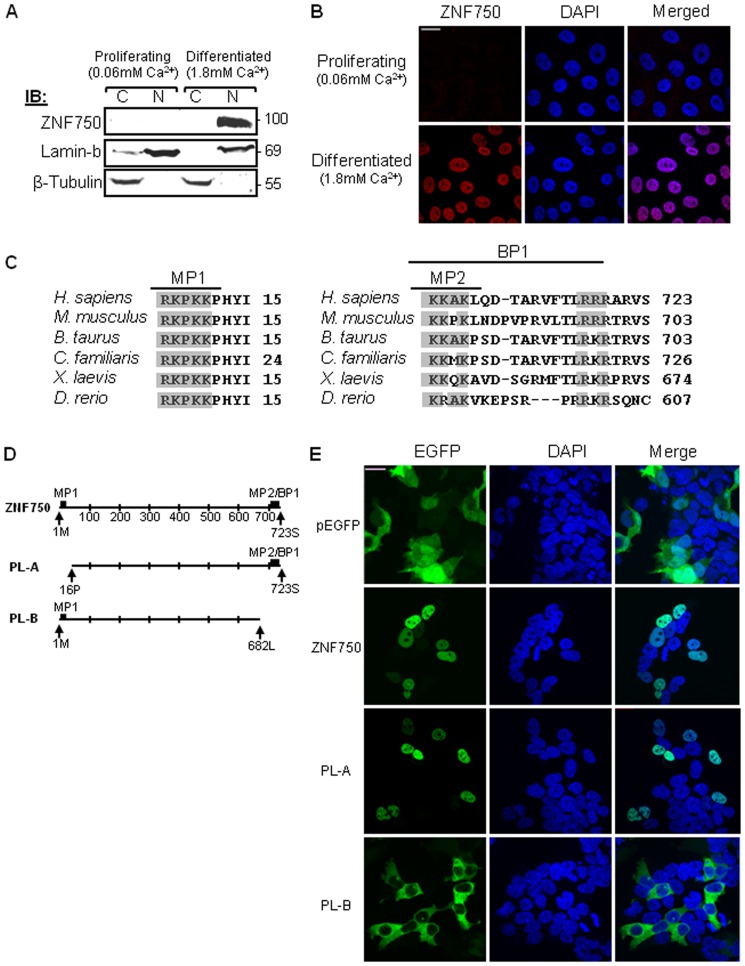
ZNF750 subcellular localization and functional NLS characterization. (A) Western blot analysis using cytoplasmic (C) and nuclear (N) fractions from HaCaT cells at proliferative/undifferentiated (0.06 mM Ca^2+^) or differentiated (1.8 mM Ca^2+^) state, analyzed by anti-ZNF750 Ab. (B) Confocal subcellular localization of ZNF750 in HaCaT cells. Cells at proliferative and differentiated states were fixed and immunostained by anti-ZNF750 Ab (red). DAPI (blue) was used to identify nuclei. Scale bar  = 20 µm. (C) Sequence alignments representing the three putative NLSs of ZNF750 homologues in various organisms. Conserved amino acids of the predicted NLSs are shaded. (D) Schematic representation of EGFP-ZNF750 constructs. (E) Confocal subcellular localization of ZNF750 constructs in HEK293 cells. The expression of pEGFP-C2 (EGFP alone) is shown as a control. EGFP (green); DAPI (blue) was used to identify nuclei. Scale bar = 20 µm.

**Figure 2:**
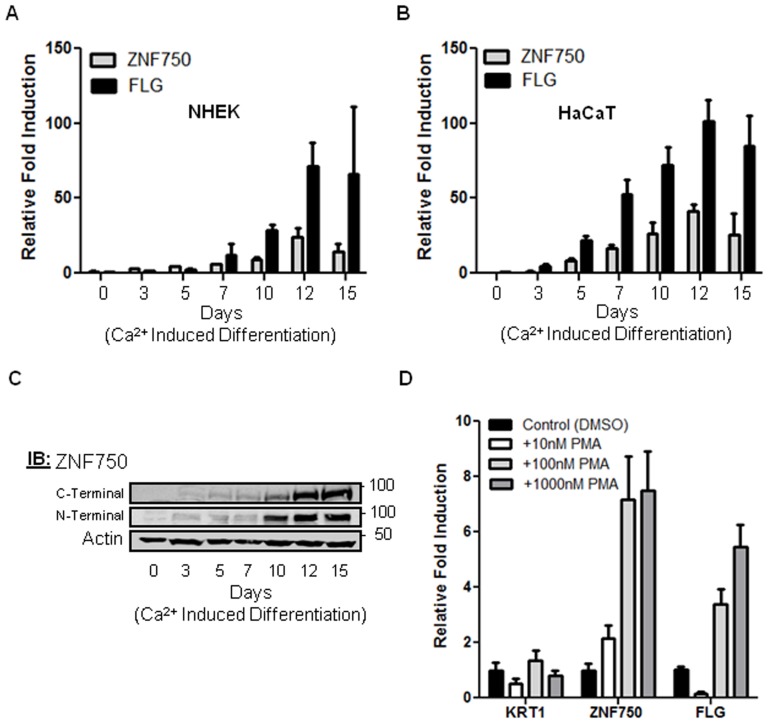
*ZNF750* mRNA and protein expression in Ca^2+^ induction and in human skin. (A) QRT-PCR of *ZNF750* mRNA expression during Ca^2+^ induction in NHEK cells. *Filaggrin* mRNA levels serve as internal control. Error bars represent mean values±SD, N = 3. (B) QRT-PCR of *ZNF750* mRNA expression during Ca^2+^ induction in HaCaT cells. Error bars represent mean values±SD, N = 3. (C) ZNF750 protein levels during Ca^2+^ induction in HaCaT cells, analyzed by anti-ZNF750 antibodies against both N-terminal and C-terminal regions. (D) QRT-PCR of *ZNF750*, *KRT1* and *FLG* mRNA expression in NHEK cells that were treated with PMA. Error bars represent mean values±SD, N = 2.

To determine the subcellular localization of ZNF750, we used western blot analysis of cytoplasmic and nuclear subcellular fractions as well as confocal microscopy with anti ZNF750 antibodies (Abs). Both assays demonstrated that ZNF750 is localized to the nucleus (Figure 1A, B). As ZNF750 is a relatively large protein of ∼100 Kilo-Daltons (figures 1A, 2C), its entry through the nuclear envelope needs to be facilitated via a heterodimeric nuclear transport receptor comprising importin α and importin β, which enable nuclear import of functional NLS-bearing proteins [10]. Bioinformatic analysis of the 723 amino-acid sequence of ZNF750 using protein domain prediction programs NLS MAPPER and PSORT II, indicated the presence of three putative highly conserved NLSs: one bi-partite (BP) NLS at the C-terminal of the molecule and two mono-partite (MP) NLSs at both the N-terminal and C-terminal regions (Figure 1C). We investigated those putative NLS regulatory motifs of ZNF750 using GFP-tagged ZNF750 (GFP-ZNF750) full length or partial length (PL) constructs (Figure 1D). Similar to the endogenous ZNF750 protein, full length GFP-ZNF750 was localized to the nucleus (Figure 1E). Partial length ZNF750 lacking the N-terminal NLS showed similar localization to that of the full length ZNF750 (PL-A, Figure 1D, E). However, removal of the ZNF750 C-terminal NLSs fully abrogated its nuclear localization (PL-B, Figure 1D, E). As per bioinformatics analysis ZNF750 has no nuclear export signal motifs, ZNF750 localization is determined and controlled by its C-terminal bi-partite NLS.

### ZNF750 Expression is Upregulated During Keratinocyte Differentiation

To determine the expression signature of *ZNF750* in KCs, we studied normal human epidermal KC (NHEK) cells and HaCaT cells [11] using Ca^2+^ induction to initiate a differentiation program which mimics the terminal differentiation that KCs undergo in vivo [12]. Through quantitative real-time PCR (QRT-PCR) and immunoblotting with Abs against both N-terminal and C-terminal regions of ZNF750, we showed that ZNF750 mRNA and protein levels increased during progression of KC differentiation in both NHEK and HaCaT cells, peaking at day 12 of Ca^2+^ induced differentiation (Figure 2A–C). Interestingly, this time line of *ZNF750* expression overlaps with that of *Filaggrin* (Figure 2A, B), a late differentiation marker of granular KCs [13]. As ZNF750 expression increased dramatically during KC differentiation (Figure 2A–C), we tested the effect of Phorbol 12-myristate 13-acetate (PMA) on *ZNF750* mRNA levels. PMA activates protein kinase C (PKC) proteins that function during terminal differentiation specifically in the transition from spinous to granular cells, inducing late differentiation markers including FLG [14]. Using QRT-PCR we measured the mRNA levels of *ZNF750* in proliferating KCs grown in low calcium media following 24 hours treatment with PMA. PMA treatment dramatically increased *ZNF750* mRNA levels in a dosage-dependent manner, similar to *FLG* expression, and had no effect on the early differentiation marker *KRT1* (Figure 2D). These in vitro findings are in line with ZNF750 studies of normal human skin sections, showing weak staining of nuclei in the suprabasal spinous layer as compared with strong staining in granular layer nuclei [15] and the sebaceous glands (Figure S1). The high expression of ZNF750 in differentiated KCs, which overlaps with that of the late differentiation marker *FLG*, suggests a role of ZNF750 in KC terminal differentiation.

### ZNF750 Knockdown in Ca^2+^ Induced HaCaT Keratinocytes Leads to Sustained Cell Proliferation and Decreased Apoptosis

**Figure 3:**
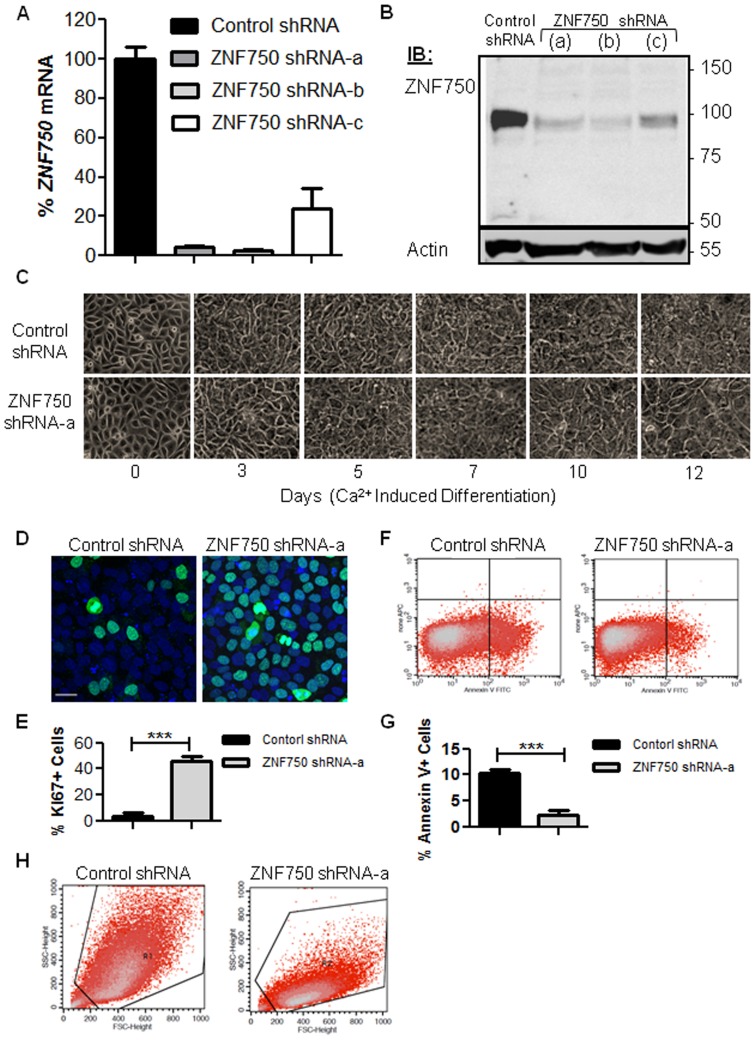
*ZNF750* silencing in HaCaT keratinocytes. HaCaT cells were transduced with scrambled shRNA (control) or with three different ZNF750 shRNAs (shRNA-a, b, and c). Cells were harvested and assayed at day 12 of Ca^2+^ induction. (A) QRT-PCR of *ZNF750* mRNA expression in the stable transduced cell lines. Error bars represent mean values±SD, N = 3. (B) western blot analysis showing ZNF750 protein levels in the stable transduced cell lines. A total of 50 µg of protein was loaded in each sample. Actin levels were measured to ensure equal amounts of loaded protein were loaded. (C) Morphological studies at different time points in HaCaT cell differentiation: control vs. *ZNF750* shRNA-a transduced cultures examined by phase contrast microscopy during Ca^2+^ induction. (D,E) *ZNF750* downregulation enhances cell proliferation. (D) Ki67 staining (green) followed by confocal microscopy. To-Pro 3 nuclear staining is shown in blue (E) quantification of Ki67 positive cells. More than 800 cells were counted for each slide, Error bars represent mean values±SD, N = 6 (***:P<0.0001). (F,G) Effects of *ZNF750* downregulation on apoptosis as assessed by Annexin V apoptosis assay. (F) Annexin V apoptosis assay using flow cytometry analysis. (G) quantification of Annexin V positive cells. More than 50,000 cells were counted in each sample Error bars represent mean values±SD, N = 4, (***:P<0.0001). (H) Effects on cell size (X axis) and granularity (Y axis) measured by flow cytometry.

Assuming a possible role of ZNF750 in KC terminal differentiation, we generated stable HaCaT cell lines in which ZNF750 expression was silenced using small hairpin RNA (shRNA). Using five different shRNA sequences targeting *ZNF750* mRNA we identified three shRNAs (shRNAa, b, and c) that achieved efficient silencing as demonstrated by both QRT-PCR and western blot analysis (Figure 3A, B). A non-targeting scrambled shRNA was used as a control. To further examine the effect of ZNF750 knockdown, cells were Ca^2+^-induced to differentiate until day twelve, when ZNF750 levels normally peak (Figure 2B, C). Compared with controls, Ca^2+^-induced HaCaT cell cultures in which *ZNF750* expression was silenced showed morphological changes compatible with impaired late differentiation (Figure 3C). We next performed a cell proliferation assay using the cell proliferation marker Ki67 [16]. ZNF750 silenced cultures sustained proliferation activity at day twelve, which was ∼10 fold higher (p<0.0001) than control cultures at that time point of late differentiation (Figure 3D,E). In addition, flow cytometry analysis of ZNF750 silenced cultures showed a ∼4 fold (p<0.0001) decrease in apoptosis, as assessed by Annexin V assay (Figure 3F, G) and markedly reduced cell granularity (Figure 3H). Thus, ZNF750 knockdown affects the in-vitro terminal differentiation program, interfering with cell cycle withdrawal.

### ZNF750 Regulates Molecular Pathways of Epidermal Terminal Differentiation

**Figure 4:**
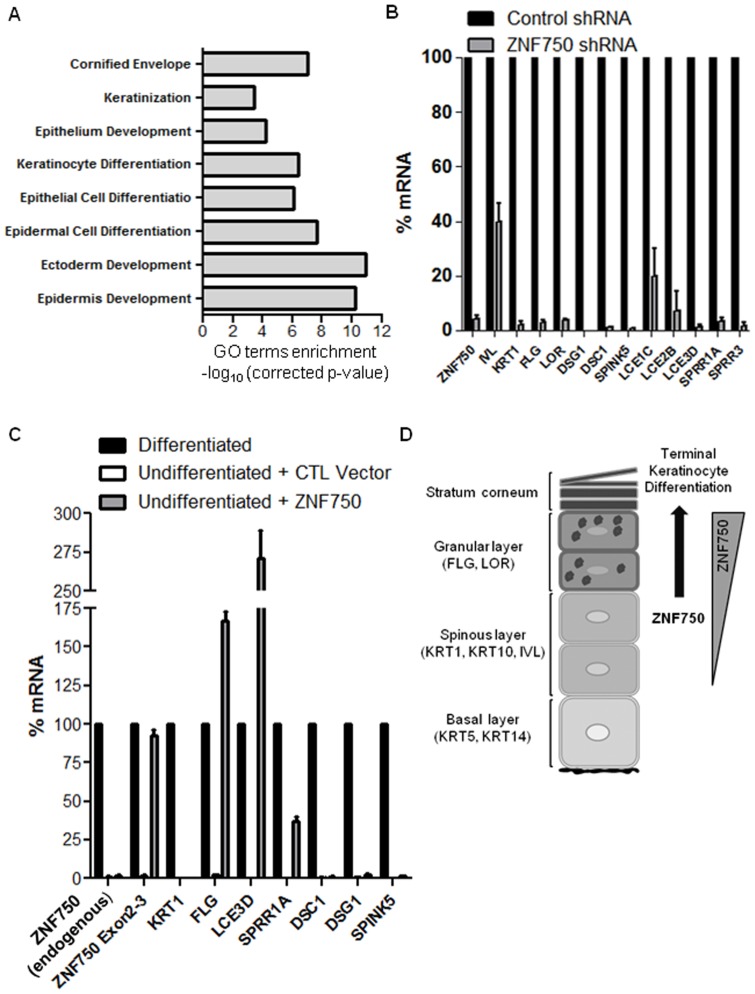
ZNF750 regulates keratinocyte terminal differentiation. (A) Gene Ontology terms for the 256 genes that were downregulated in *ZNF750* silenced cultures vs. control (P-value<0.05, FC≥2), with Bonferroni-corrected p-value<0.001. (B) QRT-PCR mRNA expression of a selected group of terminal differentiation genes in control vs. *ZNF750* silenced cultures, that showed downregulation fold change≥2. Error bars represent mean values±SD, N = 2. (C) mRNA expression (QRT-PCR) of a selected group of terminal differentiation genes in differentiated cells vs. undifferentiated HaCaT cells that overexpress *ZNF750* or an empty control vector (CTL). Cells in undifferentiated state were maintained at sub-confluence in low calcium media. Expression levels are shown relative to those of Ca^2+^-induced differentiated HaCaT cultures at day twelve (100%). Two sets of primers targeting ZNF750 were used: “ZNF750 endogenous” PCR amplifies the ZNF750 5′ UTR, representing the ZNF750 endogenous expression only, and serves as an internal control demonstrating the cells' undifferentiated state. The ZNF750 Exon2-3 primer set PCR amplifies ZNF750 coding sequence common to the endogenous and exogenous ZNF750 molecules. Note that in the CTL control group mRNA levels of all tested markers were practically null. Error bars represent mean values±SD, N = 2. (D) Schematic representation: ZNF750 expression is gradually augmented throughout keratinocyte differentiation, inducing expression of late differentiation markers.

To identify ZNF750 downstream target genes, we performed whole genome expression microarray analysis of three HaCaT cultures in which ZNF750 was silenced (triplicates of ZNF750 shRNA-a) vs. three control cultures. Cultures were Ca^2+^ induced for twelve days prior to harvesting for microarray analysis, and ZNF750 silencing was validated (Figure 3A, B). ZNF750 silencing led to significant downregulation of 256 genes and upregulation of 381 genes (Table S1, corrected p-value<0.05, fold change≥2). Genes upregulated by ZNF750 silencing showed significant enrichment of Gene Ontology[17], [18] terms related to cell cycle, cell cycle regulation, mitosis, proliferation, and cellular response to stress (corrected p-value<0.001; Figure S2). Genes downregulated by ZNF750 silencing included a subset of genes that were highly enriched in Gene Ontology terms related to KC terminal differentiation (corrected p-value<0.01; Figure 4A, Table S2). Microarray results were validated (QRT-PCR) using biological replicates (ZNF750 shRNA-a and b) for selected genes of this subset, namely *KRT1*, *INV*, *FLG*, *LOR*, *DSG1*, *DSC1*, *SPINK5*,*LCE1C*, *LCE2B*, *LCE3D*, *SPRR1A* and *SPRR3* (Figure 4B). We further examined the effects of ZNF750 overexpression in HaCaT cells. Using pHR-CMV-ZNF750 lentiviral vector, we generated a stable cell line which overexpresses ZNF750. Undifferentiated cells that were transduced with an empty vector served as a control, showing basal levels of late differentiation genes that were analyzed (Figure 4C). The undifferentiated state of the cells was confirmed by the early differentiation marker *KRT1*, and through analysis of *ZNF750* endogenous levels, using a primer set that targets the ZNF750 5′ untranslated region. Interestingly, overexpression of ZNF750 in undifferentiated KCs induced late differentiation genes that were downregulated by ZNF750 silencing, yet only ones belonging to the epidermal differentiation complex on chromosome 1q21 (Figure 4C). Thus, ZNF750 is both essential and sufficient for inducing those KC late differentiation genes.

## Discussion

Our data, together with the recent publication by Sen et al. [15], demonstrate that ZNF750 is a nuclear effector that is strongly activated in and essential for terminal KC differentiation. We showed that ZNF750 expression in the cell nucleus is determined by its highly conserved functional NLS motif within its c-terminal region. Furthermore, ZNF750 acts in terminal KC differentiation: ZNF750 is expressed in suprabasal layers, and its expression is dramatically increased in the granular layer (Figure S1), [15]. These findings are in line with our demonstration that the expression of ZNF750 increased during Ca^2+^ induction of HaCaT KC and adult primary KC differentiation in vitro, reaching maximal levels just prior to terminal KC differentiation. These findings are in line with recent findings in primary neonatal KC [15], with different kinetics likely due to different experimental systems used. Moreover, we showed that PMA, a known inducer of late differentiation markers that promotes spinous to granular transition [14], markedly induced ZNF750 expression.

ZNF750 silencing experiments further substantiated the role of ZNF750 in terminal KC differentiation: ZNF750 knockdown in Ca^2+^-induced HaCaT KCs led to arrest in the progression of late differentiation, as was evident morphologically (Figure 3C). In fact, in the silenced cells, morphological progression occurred only up to day 5–7 of in-vitro differentiation of HaCaT cells, the time point at which differentiation into spinous layer is described to be achieved at the molecular level [19]–[21], and the start point of further differentiation into granular cells. This arrest was also evident in the dramatically reduced granularity of day 12 ZNF750-silenced cells (Figure 3H). KCs in which ZNF750 was silenced demonstrated decreased apoptosis and continued proliferation into day 12 of Ca^2+^ induction. The arrested late differentiation, as evident per cell morphology at day 12, suggests that the relative enhanced proliferation of the ZNF750-silenced cells is likely due to abrogated progression into late differentiation. Our data are partly reminiscent of those seen in null mutants of Ikkα, a key regulator of KC and epidermal differentiation: Ikkα−/− mice present with a hyperproliferative and undifferentiated epidermis characterized by complete absence of a granular layer and stratum corneum [22]. Further studies are in place to unravel any molecular cascades that might link ZNF750 with Ikkα.

Molecular studies further supported the morphological findings. Using expression microarrays we demonstrated that ZNF750 knockdown depleted KC late differentiation markers such as FLG, LOR, SPINK5, SPRR3 and LCE genes, in line with similar findings recently reported by Sen et al. [15]. Many of those ZNF750 targets are mutated in various human skin diseases [2], [23]. In fact, this explains in part the clinical phenotype of the ZNF750 human mutation we previously described [8], which combines elements of the phenotypes known to emerge from mutations in some of those downstream genes. Moreover, expression of ZNF750 in undifferentiated HaCaT cells was sufficient to induce late differentiation genes, in line with similar recent findings by Sen et al. in primary keratinocytes [15]. It is interesting to note that those ZNF750-induced genes were all part of the epidermal differentiation complex (EDC), while other genes depleted by ZNF750 silencing were not induced by ZNF750 overexpression. Thus, the data provide additional evidence for a strong regulation of EDC by ZNF750. Taken together, our data suggest that ZNF750 is a regulator required for KC terminal differentiation, playing a pivotal role in this process (Figure 4D). A recent study by Sen et al. suggested that ZNF750 regulation of terminal keratinocyte differentiation is mediated by KLF4. However, overexpression of KLF4 in ZNF750 silenced keratinocytes only partially rescued expression of ZNF750-dependent terminal differentiation genes [15]. This is in line with our expression microarrays results showing that KLF4 was only slightly affected by ZNF750 silencing (failed to pass our significance terms filtering), suggesting that additional effectors that ZNF750 targets (highlighted by both studies) might mediate downstream pathways controlling terminal KC differentiation. It should be noted that the discrepancy in ZNF750-related KLF4 expression in our data as compared to the study of Sen et al. might be due to the different experimental systems used (HaCaT vs. primary KC). Further studies are warranted to determine the direct targets which mediate ZNF750 regulation of KC terminal differentiation process. Our study together with the recent findings of Sen et al. [15] highlight the essential role of ZNF750 in terminal KC differentiation, providing insights to the molecular pathways governing this process. ZNF750 and its downstream targets can serve in future elucidation of therapeutics for common diseases of impaired terminal KC differentiation and dysfunctional skin barrier.

## Materials and Methods

### Ethics Statement

We certify that the research in the manuscript “ZNF750 is expressed in differentiated keratinocytes and regulates epidermal late differentiation genes” describing immunohistochemistry studies of ZNF750 in normal human skin has been approved by the Soroka Medical Center institutional review board (IRB). No consent forms were needed as the samples were old archival samples analyzed anonymously, and most of each sample was kept for further use. The investigation approved is in accordance with the principles expressed in the Declaration of Helsinki. The Soroka Medical Center IRB waived the need for written informed consent for the use of the archival normal skin section.

### Expression Constructs

ZNF750 cDNA reference sequence was derived from GenBank (NM_024702.2). pEGFP-ZNF750 expression vector was constructed by polymerase chain reaction (PCR) amplification of ZNF750 cDNA from HaCaT cell line, utilizing the oligonucleotide primers 5′- GAATTCGTTATGAGTCTCCTCAAAGAGCGGA -3′ (forward) and 5′- GGATCCTTAGGACACCCGGGCCCTCCT -3′ (reverse), and cloning into pGEM-T (Promega, WI, USA). This was sub-cloned into the mammalian expression vector pEGFP-C2 (Clontech, CA, USA) to yield ZNF750-EGFP. In addition, two partial length (PL) constructs (PL-A and B) were generated by the use of the following primers: PL-A 5′-GAATTCCCCAGGCCTCCAGGAAAGC-3′ (forward) and 5′-GGATCCTTAGGACACCCGGGCCCTCCT-3′ (reverse). PL-B 5′-GAATTCGTTATGAGTCTCCT-3′ (forward) and 5′-GGATCCGAGGTCACACTGGGCCTCTTGGCT-3′ (reverse). PL-A (amino acids 16–723) construct abrogates ZNF750 N-terminal NLS and PL-B (amino acids 1–682) abrogates ZNF750 C-terminal NLSs.

To generate ZNF750-FLAG expression vector, ZNF750 was PCR amplified using primers: 5′-GGATCCGCCACCATGAGTCTCCTCAAAGAGCG-3′ (forward) and 5′- TCTAGATTA- CTTGTCATCGTCATCCTTGTAATCGGACACCCGGGCCCTCCT -3′ (reverse), and was sub-cloned into pHR-CMV lentiviral vector [24], to generate pHR-CMV-ZNF750 that expresses C-terminal FLAG tagged ZNF750 under the control of CMV promoter and a puromycin resistance gene via IRES sequence. All constructs were sequenced to confirm their correct sequence and orientation.

### Cell Cultures

Primary NHEK cells were purchased from Lonza (Walkersville, MD). Cells were grown in keratinocyte growth medium (KGM) containing 0.1 mM CaCl_2_ supplemented with growth factor bullet kit (Lonza, Walkersville, MD). Medium was changed every 2–3 days. Cells were used at passage 3. For differentiation, cells were cultured in KC growth media containing 1.4 mM CaCl_2_.

HaCaT cells [11], a spontaneously immortalized human KC line, were maintained in high-glucose DMEM medium containing 0.06 or 1.8 mM CaCl_2_ supplemented with 10% fetal calf serum, 1% L -glutamine, and 1% penicillin/streptomycin (Biological Industries, Beit-Haemek, Israel).

HEK293 cells were grown on a high-glucose DMEM medium containing 1.8 mM CaCl_2_ supplemented with 10% fetal calf serum, 1% L -glutamine, and 1% penicillin/streptomycin (Biological Industries).

### Lentiviral Gene Transfer

VSV-G pseudotyped single round HIV particles were produced by co-transfection of the lentiviral vector, HIV gag/pol, pRev, pTat and VSV-G, into HEK293T cells by calcium phosphate. Supernatant was harvested 48 hrs post transfection, and filtered through 0.45 µ filter. Lentiviral particles were concentrated by ultracentrifugation for 2.5 hrs at 25,000 rpm and the pellet was re-suspended in cold DMEM. HaCaT cells were transduced with viral stock that expressed the ZNF750-FLAG or a control empty vector, supplemented with 8 mg/ml polybrene, for 4 hrs. Cells were then subjected to selection with 1 µg/ml puromycin for 1 week.

### Immunohistochemistry

Formaldehyde-fixed 5-µm parffin-embedded sections of normal human skin were obtained with Approval of the Institutional Review Board of Soroka Medical Center. Sections were deparaffinized in xylene, rehydrated in decreasing concentrations of ethanol, treated with 3% H_2_O_2_ in methanol for 15 minutes at room temperature, warmed in a microwave oven in citrate buffer for 15 minutes at 90°C, and stained with rabbit polyclonal anti-ZNF750 Abs (Sigma-Aldrich, St Louis, MO) for 1 hour at room temperature. After extensive washings in phosphate-buffered saline (PBS), the antibodies were revealed using the VECTRASTAIN ABC kit (Vector Laboratories, CA, USA). To identify nuclei, slides were counterstained with hematoxylin.

### shRNA Lentiviral Transduction

We transduced HaCaT cells with *ZNF750* shRNA lentiviral particles (Table S3) or a scrambled shRNA as a control (Sigma-Aldrich) according to the manufacturer's recommendations. Briefly, 24 hours before transduction, cells were grown in six-well plates up to 5×10^4^ cells per well. Viral stock (1–10 µl) and 2 µl of 8 mg/ml polybrene were added to the cells and incubated for 18–20 hours at 37°C in a 5% CO_2_ humidified incubator. The amount of the viral stock was determined according to the desired multiplicity of infection (MOI = 5) and total transducing units (TU) per milliliter, per guidelines supplied by Sigma. At 24 hours after transduction, the cells were washed twice in 1× HBSS and maintained in DMEM containing 0.06 mM CaCl_2_. Selection was performed in the presence of puromycin (2 µg/ml) for 1 week.

### Quantitative Reverse Transcription PCR

Total RNA was isolated using TRI REAGENT (Molecular Research Center, Inc.) and subjected to DNase I (New England Bio-Labs) digestion to remove genomic DNA. RNA concentrations were determined by spectrophotometer at OD_260_. First-strand cDNA was reverse transcribed from 500 ng of RNA by the Reverse-iT kit (ABgene, Epson, UK) using oligo-dT primers. cDNA PCR amplification was carried out using the SYBR Green JumpStart Taq ReadyMix (Sigma-Aldrich) on a Rotor-Gene RG-3000 real-time PCR detection system (Corbett Research) with gene-specific intron-crossing oligonucleotide pairs listed in Table S4. To ensure the specificity of the reaction conditions, at the end of the individual runs, the melting temperature (Tm) of the amplified products was measured to confirm its homogeneity. Cycling conditions were as follows: 95°C for 10 minutes, 95°C for 20 seconds, 60°C for 15 seconds, and 72°C for 20 seconds for a total of 40 cycles. Each sample was analyzed in triplicate. Results were normalized to *GAPDH* mRNA levels. After the quantification procedure, the products were separated on 2% agarose gel electrophoresis to confirm that the reaction had amplified DNA fragments of expected size.

### Expression Microarray Hybridization and Data Analysis

Microarray analysis was performed on biological triplicate samples. Labeled cRNA was hybridized to Affymetrix GeneChip HuGene-1_0-st-v1 arrays. Data have been deposited in GEO database compliance with MIAME guidelines. For gene expression analysis, arrays were RAM normalized [25] and differential expression was defined using the following filters: Statistical hypothesis testing for identification of differentially expressed genes was done using 2-Way ANOVA in Partek. P-values were adjusted for multiple testing by step-up FDR [26]. Fold change values are represented on linear scale, where positive and negative values indicate up- and down-regulation, respectively. Differentially expressed genes were defined as those having absolute expression signal >5 (log2 scale) in at least one of the arrays. Results were considered significant for FDR adjusted p-value<0.05 in any of the effects or interaction, and an average fold change ≥2. Gene Ontology term enrichment was performed using DAVID [17], [18], and p-values represent a Bonferroni corrected p-value.

### Subcellular Fractionation and Western Blot Analyses

Cells were washed with 1× PBS two times before addition of the cytoplasmic lysis buffer (10 mM Hepes at pH 8.0, 1.5 mM MgCl_2_, 10 mM KCl, 0.5 mM DTT, 300 mM sucrose, 0.1% NP-40, 10 mM NaF, 10 mM Na 3 VO_4_, 1× protease inhibitors, 0.5 mM PMSF). Cells were lysed for 10 min on ice and then quick-spun for 15 sec to collect cytosolic lysate. Pellets were washed two times with cytoplasmic lysis buffer and then lysed with nuclear lysis buffer (50 mM Hepes at pH 7.9, 250 mM KCl, 0.1 mM EDTA, 0.1 mM EGTA, 0.1% NP-40, 0.1% glycerol, 10 mM NaF, 10 mM Na_3_VO_4_ , 1 mM DTT, 1× protease inhibitors, 0.5 mM PMSF) for 30 min on ice. The lysates were spun for 20 min at 14,000 rpm at 4°C to collect nuclear lysates.

Cell lysates (50 µg) were used for immunoblotting, electrophrated on 8% SDS-PAGE and transferred to nitrocellulose membranes. Membranes were incubated in primary antibodies for 1 hr each. The primary Abs included Lamin-B1, β-Tubulin (Santa Cruz Biotechnology, Santa Cruz, CA) and ZNF750 (Sigma-Aldrich). After incubation with secondary horseradish peroxidase conjugated anti-goat or anti-rabbit antibodies (Santa Cruz Biotechnology), proteins were detected using the EZ-ECL chemiluminescence detection kit (Biological Industries). To compare the amount of protein in different samples, the blots were re-probed using anti β-actin Ab (Santa Cruz Biotechnology) and secondary horseradish peroxidase-conjugated anti-goat Ab (Santa Cruz).

### Confocal Microscopy

HaCaT cells were cultured on glass coverslips to 60–80% confluence in media containing 0.06 mM or 1.8 mM CaCl2. Cells were washed with 1× PBS, fixed in 4% paraformaldehyde, permeabilized using Triton X-100 (0.5% v/v), incubated with primary rabbit anti-ZNF750 (Sigma-Aldrich) and secondary goat anti-rabbit AlexaFluor 546 (Invitrogen Corporation, CA,USA) antibodies, and then mounted with Vectashield (Vector Laboratories). HEK293 cells were cultured on a glass coverlips to 50–60% confluence, and transiently transfected with 1 µg DNA of an expression construct using lipofectamin2000 (Invitrogen). At 24 hours post transfection, cells were washed twice with 1× PBS, fixed in 4% paraformaldehyde, and permeabilized using Triton X-100 (0.5% v/v) and mounted with Vectashield (Vector Laboratories). The subcellular localizations were visualized using an Olympus confocal microscope with an ×40 and ×60 objective. Confocal images were recorded under identical conditions. Excitation was performed with a 488 nm (for EGFP), 504 nm (for DAPI) and a 543 nm (for AlexaFluor 546) laser and filtered accordingly.

### Ki67 Proliferation Assay

HaCaT cells with stable expression of ZNF750 shRNA or a control shRNA were cultured on glass coverslips to 80–90% confluence in media containing 0.06 mM CaCl_2_. Cells were induced to differentiate in media containing 1.8 mM CaCl_2_. At day twelve of calcium induced differentiation, cells were washed twice with 1×PBS and fixed in 4% paraformaldehyde. Prior to staining, primary antibody diluting buffer (Biomeda Corp., Foster City, CA), containing Triton X-100 (0.5% v/v), was used to block nonspecific binding. Cells were incubated with rabbit anti Ki67 primary antibody (Cell Marque, Rocklin, CA) for 1 hour, followed by secondary goat anti rabbit AlexaFluor 488 (Invitrogen). To-Pro 3 (Molecular Probes, Invitrogen, USA) was used for nuclear staining. Cells were examined under an Olympus Fluoview FV1000 confocal laser scanning microscope using ×20 or ×60 objectives. Confocal images were recorded under identical conditions. Excitation was performed with a 488 nm (for AlexaFluor 488), and a 504 nm (for DAPI) laser and filtered accordingly. Ki67 positive cells were counted as a percent of total cells using Cell Profiler software. More than 800 cells were counted in each slide. N = 6 Differences in proliferation activity were considered significant at P-values<0.0001 calculated using a standard student's t-test.

### Annexin V Assay

Annexin V assay was performed using MEBCYTO Apoptosis Kit (MBL CO., LTD. Nagoya, Japan) according to the manufacturer's protocol. Briefly, cells were incubated with Annexin V at room temperature for 15 minutes in the dark and then subjected to flow cytometry analysis. More than 50,000 cells were counted each time, N = 4. Differences in apoptotic activity were considered significant at P-values<0.0001, calculated using standard student's t-test.

### Accession Numbers

Microarray data have been deposited into the GEO database with accession number GSE38039.

## Supporting Information

Table S1 **Genes that are upregulated or downregulated 2-fold or more in** ***ZNF750*** **silenced HaCaT cultures at day twelve of Ca^2+^ induction.**
(XLSX)

Table S2 **Gene Ontology terms of selected ZNF750 target genes that were downregulated by ZNF750 silencing.**
(PDF)

Table S3 **shRNA sequences used for** ***ZNF750*** **silencing**.
(PDF)

Table S4 **Oligonucleotide sequences used in quantitative real-time PCR**.
(PDF)

Figure S1 **ZNF750 immunohistochemistry staining in normal human skin.**
(PDF)

Figure S2 **Genes whose expression was upregulated by ZNF750 silencing in HaCaT cells: Gene Ontology (GO) terms enrichment.**
(PDF)
