# Supplementary material for: ZNF750 Is Expressed in Differentiated Keratinocytes and Regulates Epidermal Late Differentiation Genes
Source: PLoS One. 2012 Aug 24;7(8):e42628. doi: 10.1371/journal.pone.0042628 (PMC3427353; doi:10.1371/journal.pone.0042628)
Supplement: Table S2 — Gene Ontology terms of selected ZNF750 target genes that were downregulated by ZNF750 silencing. (PDF) [file pone.0042628.s002.pdf]

Table S2. Gene Ontology terms of selected ZNF750 target genes that were downregulated by ZNF750 silencing.

Symbol	Gene Name	Fold Change	Gene Ontology term
CSTA	cystatin A (stefin A)	-4.40697	Cornified Envelope
FLG	filaggrin	-14.4208	Cornified Envelope
HRNR	hornerin	-35.1653	Cornified Envelope
RPTN	repetin	-37.029	Cornified Envelope
SCEL	sciellin	-2.60339	Cornified Envelope
SPRR1A	small proline-rich protein 1A	-14.1643	Cornified Envelope
SPRR1B	small proline-rich protein 1B (cornifin)	-4.37015	Cornified Envelope
SPRR2A	small proline-rich protein 2A	-4.50311	Cornified Envelope
SPRR2E	small proline-rich protein 2E	-3.90093	Cornified Envelope
SPRR3	small proline-rich protein 3	-27.6549	Keratinocyte Differentiation
LCE3D	late cornified envelope 3D	-2.19341	Keratinocyte Differentiation
KRT2	keratin 2	-3.38804	Keratinocyte Differentiation
S100A7	S100 calcium binding protein A7	-13.66	Keratinocyte Differentiation
ALOX12B	arachidonate 12-lipoxygenase, 12R type	-3.40385	Epidermis Development
CALML5	calmodulin-like 5	-6.46431	Epidermis Development
CASP14	caspase 14, apoptosis-related cysteine peptidase	-22.7243	Epidermis Development
FABP5	fatty acid binding protein 5 (psoriasis-associated)	-7.21701	Epidermis Development
KLK14	kallikrein-related peptidase 14	-2.036	Epidermis Development
KRT1	keratin 1	-5.00053	Epidermis Development
KRT10	keratin 10	-2.42125	Epidermis Development
KRT16	keratin 16	-2.56328	Epidermis Development
SPINK5	serine peptidase inhibitor, Kazal type 5	-19.5388	Epidermis Development
TGM5	transglutaminase 5	-6.1669	Epidermis Development
DLX5	distal-less homeobox 5	-2.95434	Epithelial cell Differentiation
UPK2	uroplakin 2	-2.2658	Epithelial cell Differentiation
KRT3	keratin 3	-4.64879	Epithelial Development
WNT4	wingless-type MMTV integration site family, member 4	-2.04847	Epithelial Development
