# Supplementary material for: ZNF750 Is Expressed in Differentiated Keratinocytes and Regulates Epidermal Late Differentiation Genes
Source: PLoS One. 2012 Aug 24;7(8):e42628. doi: 10.1371/journal.pone.0042628 (PMC3427353; doi:10.1371/journal.pone.0042628)
Supplement: Table S3 — shRNA sequences used for ZNF750 silencing. (PDF) [file pone.0042628.s003.pdf]

Table S3. shRNA sequences used for *ZNF750* silencing.

shRNA	Sequence
ZNF750 shRNA-a	5’CCGGGAAAGCCCTTCAAGTATAAATCTCGAGATTTATACTTGAAGGGCTTTCTTTTTG 3’
ZNF750 shRNA-b	5’CCGGATTACTGGTGAAGGTATTATCCTCGAGGATAATACCTTCACCAGTAATTTTTTG 3’
ZNF750 shRNA-c	5’CCGGGAGTTCCCAAGTGCCCTAAATCTCGAGATTTAGGGCACTTGGGAACTCTTTTTG 3’
