# Supplementary material for: ZNF750 Is Expressed in Differentiated Keratinocytes and Regulates Epidermal Late Differentiation Genes
Source: PLoS One. 2012 Aug 24;7(8):e42628. doi: 10.1371/journal.pone.0042628 (PMC3427353; doi:10.1371/journal.pone.0042628)
Supplement: Table S4 — Oligonucleotide sequences used in quantitative real-time PCR. (PDF) [file pone.0042628.s004.pdf]

Table S4. Oligonucleotide sequences used in quantitative real-time PCR.

Gene Symbol	Forward primer	Reverse primer
DSG1	ATCCAGCAGAGATGGACTGG	GTTTCTGCCTTCGGATTGAA
FLG	GGCAAATCCTGAAGAATCCA	TGCTTTCTGTGCTTGTGTCC
GAPDH	TCGACAGTCAGCCGCATC	CCGTTGACTCCGACCTTC
IVL	TCTGCCTCAGCCTTACTGTG	CAGTGGAGTTGGCTGTTTCA
KRT1	TCTCGGTTGGATTCGGAACTGAAG	AGACAACTCTGCTTGGTAGAGTGC
LCE1C	GCTGAAGGACCCTGTGCTG	ACACTTTGGGGGACACTTTG
LCE2B	GGTTGACTAAACTCTGCCAGG	CACTGGGGCAGGCATTTA
LCE3D	CCTTCTCCTGCCTCCTCTG	CACTTGGGTGAGGGACACTT
LOR	GAGTAGCCGCAGCCAGAAC	CTCCTCACTCACCCTTCCTG
SPINK5	GACATCTAAGAGTACAGCTTCCTT	TGTTGCCATGCATTTTCCCATCTG
SPRR1A	GACCACACAGCCCATTCTG	CTCCTTGGTTTTGGGGATG
SPRR3	GCTGAACACCTCGACCTTCT	TTCAAAGGATGCTGGACACA
ZNF750 (endogenous)	CAGGTACTGCTTCCTGAGCAC	GAGAGCCTCCGTCATCTGG
ZNF750 Exon2-3	GCCTTCAGGCCTGTTAAGAA	GGCTGCCAGGTTTATCTCTG
