# Supplementary material for: ZNF750 Is Expressed in Differentiated Keratinocytes and Regulates Epidermal Late Differentiation Genes
Source: PLoS One. 2012 Aug 24;7(8):e42628. doi: 10.1371/journal.pone.0042628 (PMC3427353; doi:10.1371/journal.pone.0042628)
Supplement: Figure S1 — ZNF750 immunohistochemistry staining in normal human skin. (PDF) [file pone.0042628.s005.pdf]

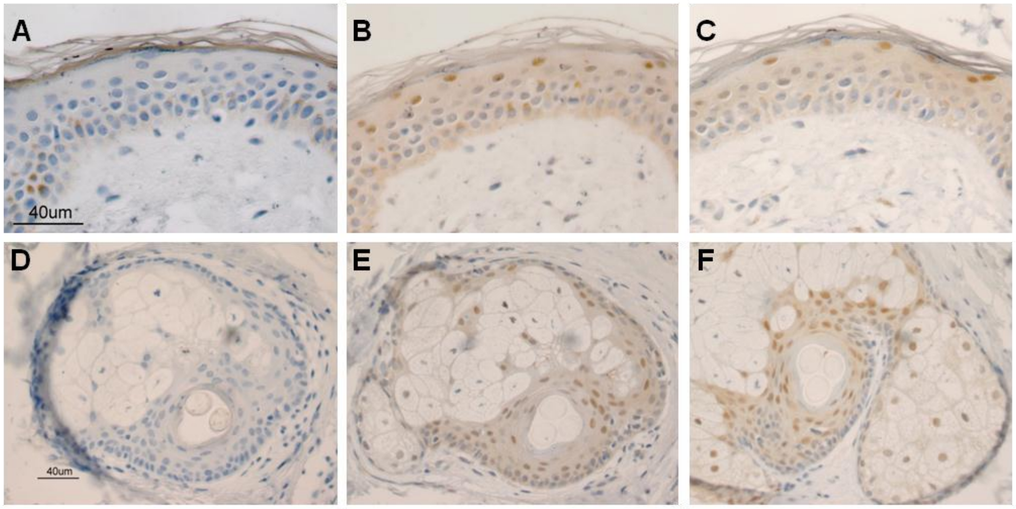

Figure S1. ZNF750 immunohistochemistry staining in normal human skin.

ZNF750 protein immunohistochemistry staining of normal human skin sections. (A-C) Staining of dermis and epidermis. (D-F) staining of sebaceous glands. (A, D) Negative control without primary antibody. (B, E) Anti-ZNF750 antibodies targeted at the C-terminal region of ZNF750. (C, F) Anti-ZNF750 antibodies targeted at the N-terminal region of ZNF750. To identify nuclei, slides were counterstained with hematoxylin. Note that ZNF750 is absent in dermal fibroblasts. Bar=40μm.
