# Supplementary material for: ZNF750 Is Expressed in Differentiated Keratinocytes and Regulates Epidermal Late Differentiation Genes
Source: PLoS One. 2012 Aug 24;7(8):e42628. doi: 10.1371/journal.pone.0042628 (PMC3427353; doi:10.1371/journal.pone.0042628)
Supplement: Figure S2 — Genes whose expression was upregulated by ZNF750 silencing in HaCaT cells: Gene Ontology (GO) terms enrichment. (PDF) [file pone.0042628.s006.pdf]

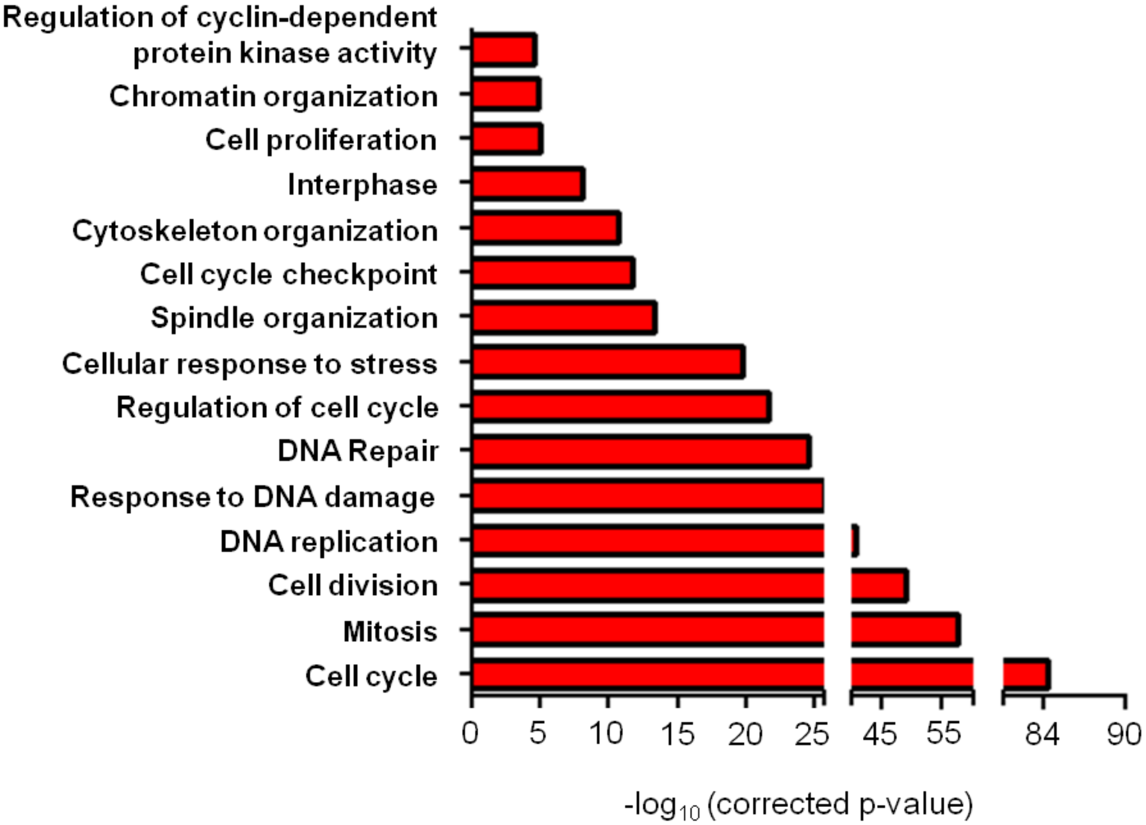

Figure S2. Genes whose expression was upregulated by ZNF750 silencing in HaCaT cells: Gene Ontology (GO) terms enrichment.

For the 381 genes that were upregulated in ZNF750 silenced cultures (shRNA-a) vs. controls (p-value<0.05, FC>2), a total of 59 GO terms were significantly enriched (Bonferroni-corrected p-value<0.001). Selected GO terms are shown.
